# Comparative Transcriptome Profiling of mRNA and lncRNA Related to Tail Adipose Tissues of Sheep

**DOI:** 10.3389/fgene.2018.00365

**Published:** 2018-09-10

**Authors:** Lin Ma, Meng Zhang, Yunyun Jin, Sarantsetseg Erdenee, Linyong Hu, Hong Chen, Yong Cai, Xianyong Lan

**Affiliations:** ^1^Shaanxi Key Laboratory of Molecular Biology for Agriculture, College of Animal Science and Technology, Northwest A&F University, Yangling, China; ^2^Key Laboratory of Adaptation and Evolution of Plateau Biota, Northwest Institute of Plateau Biology, Chinese Academy of Sciences, Xining, China; ^3^Science Experimental Center, Northwest University for Nationalities, Lanzhou, China; ^4^College of Life Science and Engineering, Northwest University for Nationalities, Lanzhou, China

**Keywords:** sheep, transcriptome, fat deposition, long non-coding RNA (lncRNA), fat tail

## Abstract

The Lanzhou Fat-Tail sheep (LFTS, long fat-tailed sheep) is an endangered sheep breed in China with a fat tail compared to the traditional local varieties, Small Tail Han sheep (STHS, thin-tailed sheep) with a small tail, and Tibetan sheep (TS, short thin-tailed sheep) with a little tail. However, little is known regarding how tail fat deposition is regulated by long noncoding RNA (lncRNA). To evaluate the lncRNA and mRNA associated with tail fat deposition and development among these breeds, high-throughput RNA sequencing of three individuals each of LFTS, STHS, and TS were performed and analyzed in this study. RNA sequencing data from these three groups revealed 10 differentially expressed genes (DEGs) and 37 differentially expressed lncRNAs between the LFTS and STHS groups, 390 DEGs and 59 differentially expressed lncRNAs between the LFTS and TS groups, and 80 DEGs and 16 differentially expressed lncRNAs between the STHS and TS groups (*p*-value < 0.05 and fold change ≥ 2), respectively. Gene Ontology and pathway analysis of DEGs and target genes of differentially expressed lncRNAs revealed enrichment in fatty acid metabolism and fatty acid elongation-related pathways that contribute to fat deposition. Subsequently, the expression of 14 DEGs and 6 differentially expressed lncRNAs was validated by quantitative real-time PCR. Finally, two co-expression networks of differentially expressed mRNA and lncRNAs were constructed. The results suggested that some differentially expressed lncRNAs (*TCONS_00372767, TCONS_00171926, TCONS_00054953*, and *TCONS_00373007*) may play crucial roles as core lncRNAs in tail fat deposition processes. In summary, the present study extends the sheep tail fat lncRNA database and these differentially expressed mRNA and lncRNAs may provide novel candidate regulators for future genetic and molecular studies on tail fat deposition of sheep.

## Introduction

Lanzhou Fat-Tailed sheep (LFTS), Small Tailed Han sheep (STHS), and Tibetan sheep (TS) are famous and special sheep breeds in China. LFTS are one of the four Chinese sheep breeds majorly raised in Northwestern China where the terrain is dry and the region is at high altitude. However, the famous phenotype of LFTS is their fat tail, which can sag to the hock and accumulate a lot of fat (Shelton, 1990; Almeida, 2011; Edea et al., 2017; Li et al., 2018a). Currently, the number of fat-tailed sheep are in sharp decline, especially LFTS. LFTS is an endangered breed that needs protection. Compared with LFTS, STHS have smaller tails and fat accumulation (Xu et al., 2017; Ma et al., 2018). STHS have a high reproductive capacity and show polyembryony; they grow fast and could be in oestrum at all seasons (Kashan et al., 2005). TS are raised in the mountainous region of the Qingzang plateau, where the average elevation is 3,500 m. Compared with LFTS and STHS, TS are relatively stronger and their tails are the smallest with less fat accumulation (Zhu et al., 2016; Zhou et al., 2017).

Adipose tissue is one of the vital tissues involved in the regulation of fat development and lipid metabolism in domestic animals. The “fat-tail” can provide energy during migration and in seasons when the pasture is dormant or when low amounts of dry matter are available (Atti et al., 2004). The fat-tail phenotype is a trait necessary for survival in harsh environments (Pourlis, 2011). In addition, the tail fat of sheep can be used by humans as an important source of dietary fat (Kashan et al., 2005; Moradi et al., 2012). Thus, the mechanism of tail fat deposition is worth studying.

In recent years, deep sequencing of transcriptomes is increasingly being utilized with promises of higher sensitivity in identification of differential expression (Jäger et al., 2011; Miao and Luo, 2013; Zhang C. et al., 2013). A few comparative transcriptome studies and whole genome studies were performed to survey gene expression profiles between different sheep breeds and different tissues in the same sheep breed (Wang et al., 2014; Miao et al., 2015b; Kang et al., 2017; Zhi et al., 2017). There are some studies on miRNA or CNV in the adipose in sheep (Miao et al., 2015a; Zhu et al., 2016; Zhou et al., 2017). In 2014, transcriptome sequencing was used to compare transcriptome profiles of fat between a fat-tailed sheep (Kazak sheep) and a short-tailed sheep (TS). 646 genes were differentially expressed between the two breeds, and the two top genes with the largest fold change (*NELL1* and *FMO3*) may affect fat metabolism in adipose tissues of sheep (Wang et al., 2014). In 2015, 602 differentially expressed genes (DEGs) were identified in the fat of two breeds of sheep using RNA-Seq technology, and some of these genes were shown to be involved in fat metabolism process through GO enrichment and KEGG pathway analysis. These genes may be involved in fat deposition in sheep (Miao et al., 2015b). The miRNA were sequenced in fat of two breeds of sheep and 54 differentially expressed miRNA were identified. It was found that some miRNA and their target genes were involved in the tail lipid development of sheep. (Miao et al., 2015a). In 2017, deep sequencing methods were used to identify miRNA and their target genes involved in the fat of the fat-tailed sheep (Kazakhstan sheep) and thin-tailed sheep (TS). By comparing the HiSeq data of these two breeds, it was found that some miRNA were involved in the development of tail fat, and through the integration analysis of miRNA–mRNA, it is revealed that some miRNA and their target genes play a key role in fat deposition in sheep (Zhou et al., 2017). In the same year, 1,058 DEGs were identified by transcriptome sequencing of three different types of fat (subcutaneous fat, visceral fat, and tail fat) in Tan sheep, and it was suggested that *HOXC11, HOXC12, HOXC13, HOTAIR_2, HOTAIR_3*, and *SP9* could be associated with tail fat deposition in sheep (Kang et al., 2017). Recently, transcriptome sequencing and miRNA sequencing were performed in three types of fat (subcutaneous fat, perirenal fat, and tail fat) of two sheep breeds (Guangling large-Tailed sheep and Small-Tailed Han sheep). Fat-related genes (*FABP4, FABP5, ADIPOQ*, and *CD36*) were highly expressed, and 14 genes (*LOC101102230, PLTP, C1QTNF7, OLR1, SCD, UCP-1, ANGPTL4, FASD2, SLC27A6, LAMB3, LAMB4, RELN, TNXB*, and *ITGA8*) and 9 miRNA (miR-10b, miR-29a, miR-30c, miR-155, miR-192, miR-206, novel-miR-102, novel-miR-36, and novel-miR-63) may be associated with fat deposition in sheep (Li et al., 2018b; Pan et al., 2018). However, up to now, there has been no report on long non-coding RNAs (lncRNAs) of the fat tail in sheep. Furthermore, more complex gene networks and molecular determinants related to tail fat development remain unclear and further studies exploring these aspects are required.

Here, in order to characterize the mRNA and lncRNA expression profiles in the tail fat of sheep, we explored the transcriptomic differences among LFTS, STHS, and TS sheep and elucidated the molecular mechanisms of tail fat deposition. Our study may provide more clues from coding and non-coding regions regarding the mechanism of fat deposition in fat-tailed sheep.

## Materials and Methods

### Ethics Statement

All experiments performed in this study were approved by the International Animal Care and Use Committee of the Northwest A&F University (IACUC–NWAFU). Furthermore, the care and use of animals complied with the local animal welfare laws, guidelines, and policies.

Experimentallicense on the basis of “Experimental Animal Management Measures in Shaanxi Province” (016000291szfbgt-2011-000001), all experiment procedures, were approved by the Review Committee for the Use of Animal Subjects of Northwest A&F University. Animal experimentation, including sample collection, was performed in agreement with the ethical commission’s guidelines. This license is for LM, etc., thesis on “Comparative transcriptome profiling of mRNA and lncRNA related to tail adipose tissues of sheep.” College of Animal Science and Technology, Northwest A&F University, Yangling, Shaanxi, China, January 26, 2018.

### Animal and Tail Fat Tissue Collection

In this study, nine unrelated individuals of LFTS (*n* = 3), STHS (*n* = 3), and TS (*n* = 3) breeds that were castrated at the age of 6 months were randomly selected from a sheep farm located in Gansu province, China. The appearance and shape of the sheep completely conformed to their varietal characteristics. Their body conditions were healthy and their weights were moderate. The sheep were fed in stables under natural lighting. The animals were slaughtered and the tail fat tissues collected. The fresh tissues were immediately frozen in liquid nitrogen, and then stored at -80°C until use.

### RNA Extraction and Quality Assessment

Total RNA was extracted from tail fat tissues using RNAiso Plus (Takara, Dalian, China) following the manufacturer’s specifications. The RNA was, respectively, solubilized in 30 μL DEPC-treated H_2_O. Aliquots of 1 μL RNA from each sample were used for evaluation by spectrophotometric analysis. Another aliquot of 1 μL RNA mixed in loading buffer was detected on 1.0% agarose gel electrophoresed for 20 min by staining with ethidium bromide and observing under UV transillumination. The RNA concentration and quality were assessed by the Agilent 2100 bioanalyzer (Agilent Technologies, Santa Clara, CA, United States). The *A*_260/280_ ratios, 28S/18S values, and the RNA Integrity Numbers (RIN) of all samples are shown in **Supplementary Table S1**. Subsequent sequencing experiments were performed on qualified RNA. The remaining RNA samples were immediately stored at -80°C.

### cDNA Library Construction and Illumina Sequencing

Qualified total RNA was further purified by RNAClean XP Kit (Beckman Coulter, Inc., Kraemer Boulevard, Brea, CA, United States) and RNase-Free DNase Set (Qiagen, GmBH, Germany). After the purification and ribosomal RNA removal, the rRNA-depleted samples were sheared into small fragments using divalent cations under high temperature. These RNA fragments were copied into the first strand of cDNA using random primers and reverse transcriptase. The second strand of cDNA was then synthesized using DNA Polymerase I and RNase H. These final cDNA fragments were then subjected to an end repair process where a single “A” base was added followed by ligation of the adapters. The output was then purified and enriched using PCR to create the final cDNA library.

The nine strand-specific RNA-Seq libraries were sequenced with a HiSeq 2000 Desktop Sequencer from Illumina Sequencing Technologies (Biotechnology, Shanghai, China). Sequencing was optimized to generate 150 bp paired reads. All datasets have been submitted to NCBI Sequence Read Archive (SRA) database and the files can be found under the accession numbers SRR6666247, SRR6666246, SRR6666245, SRR6666244, SRR6666251, SRR6666250, SRR6666249, SRR6666248, SRR6666243.

### Sequencing Quality Assessment, Reads Mapping, and Transcriptome Assembly

Reads qualities of the RNA sequencing (RNA-Seq) were evaluated using FastQC (v0.10.1) (Andrews, 2012). Adaptor sequences and low quality sequences were removed from the original reads by Seqtk^1^. The clean reads for each sample were mapped to the sheep reference genome *Ovis aries v3.1* with TopHat2 (v2.0.9) using the paired-end mapping method with two mismatches (Trapnell et al., 2009). Based on it, the transcripts were assembled using Cufflinks (v2.2.1) with default parameters (Trapnell et al., 2012).

### Prediction of lncRNA

After annotation, the unknown transcripts were used to screen for lncRNA candidates. Transcripts smaller than 200 nucleotides or having single exons were discarded. Based on the length of the open reading frame, homology with known proteins and their coding potential, the Coding Potential Calculator (Kong et al., 2007), the Coding-Non-Coding Index (Sun et al., 2013), and the Protein Families Database (Finn et al., 2014), which have the power to sort lncRNAs from putative protein-coding RNAs were combined to screen the lncRNAs. The transcripts from the intersection of the three methods were predicted to be lncRNA transcripts.

### Screening of DEGs and Differentially Expressed lncRNAs

DEGs were analyzed by edgeR package to calculate the *p*-value that was obtained by multiple hypothesis testing calibration (Robinson et al., 2010). The *p*-value was corrected using the false discovery rate (FDR) to obtain the *q*-value. *Q*-values were then used to calculate the differential expression among the three groups.

We also calculated fragments per kilobase of the exon model per million mapped reads (FPKM) value of each gene using Perl script, as follows:

$$\text{FPKM}=\frac{\text{total exon fragments}}{\text{mapped reads (Millons) }\times \text{ exon length (KB)}}$$

FPKM were used to calculate the fold change of DEGs among the three groups. Differentially expressed lncRNAs were analyzed by Cuffdiff to calculate the *q*-value and fold change (Trapnell et al., 2012). Transcript abundance of lncRNAs was measured by FPKM using Cufflinks (v2.2.1) (Trapnell et al., 2012). DEGs or differentially expressed lncRNAs with a *q*-value < 0.05 and an absolute value of fold change ≥ 2 were assigned as differential expression. Based on the FPKM of all genes or lncRNAs from three groups of pairwise comparisons, the volcano were plotted by gglot2 packages to show the patterns of genes/lncRNAs expression.

### Target Gene Prediction

Differentially expressed lncRNAs were selected for target prediction via *cis*- or *trans*-regulatory effects. For the *cis* pathway target gene prediction, the genes transcribed within a 10-kb window upstream or downstream of lncRNAs were considered as *cis* target gene. RNAplex software was then used to select *trans*-acting target genes (Tafer and Hofacker, 2008).

### Gene Ontology (GO) and Kyoto Encyclopedia of Genes and Genomes (KEGG) Pathway Analyses of DEGs and Target Genes of Differentially Expressed lncRNAs

To analyze the main function of the genes and lncRNAs, DEGs and the target genes were annotated through the GO and KEGG. The GO database was used to predict and illuminate the function of the gene product with respect to the molecular and biological processes and cellular component (Ashburner et al., 2000). The genes were first mapped to the GO terms in the database^2^. The gene numbers in every GO term were then calculated to determine the significantly enriched GO terms using the corrected *p*-value < 0.05 as a threshold. KEGG^3^ was used to perform pathway enrichment analysis (Kanehisa et al., 2016) to confirm the main biochemical and signaling pathways in which the genes participate. The significantly enriched KEGG pathways were determined using the corrected *p*-value < 0.05 as a threshold. If the corrected *p*-value (*q*-value) < 0.05, significant enrichment of GO terms, or KEGG pathways was observed in the DEGs and target genes of differentially expressed lncRNAs.

### Validation of RNA-Seq Results by Quantitative Real-Time PCR (qRT-PCR)

To quantitatively determine the reliability of our analyzed data, 14 significant DEGs and 6 differentially expressed lncRNAs were randomly selected to test their expression levels using qRT-PCR. Total RNA samples were reverse transcribed to cDNA using the PrimeSript^TM^ RT reagent Kit with gDNA Eraser (TaKaRa, Dalian, China) according to the manufacturer’s recommendations. qRT-PCR was performed using the SYBR^®^ Premix Ex Taq^TM^ kit (TaKaRa, Dalian, China) on the Bio-Rad CFX96 Real-Time PCR system (Hercules, CA, United States). All the primers of DEGs and differentially expressed lncRNAs used are presented in **Tables 1**, **2**, respectively. Individual samples were run in triplicate. The qRT-PCR amplification program was as follows: pre-denaturation at 95°C for 30 s, followed by 39 cycles of 95°C for 5 s, 60°C for 30 s.

**Table 1:** Primer pairs of DEGs used for qRT-PCR validation.

Gene	Primer (5^′^→3^′^)	Product size (bp)	Primer position
*FMO2*	F: CAGGTATCCAGAAGTTCAAA	105	ENSOART00000013523.1: 488–507
	R: CTGAGTTTCCTATTCCAATCA		ENSOART00000013523.1: 572–592
*PENK*	F: TGGGAGATGAAACCAAAGAG	171	ENSOART00000021977.1: 446–465
	R: CAGGAACTTCCTTGGAGTAA		ENSOART00000021977.1: 597–616
*DPT*	F: GAGTGGCAATTTTACTGCTG	132	ENSOART00000010048.1: 379–398
	R: CCCTCGCATATAATAATCATAATTG		ENSOART00000010048.1: 486–510
*RASD1*	F: CTACCAACTGGACATCCTC	190	ENSOART00000001878.1: 421–439
	R: CTCCTTGGTCTTGTTCTTTAG		ENSOART00000001878.1: 590–610
*MID1IP1*	F: CGACACCTACAACCAGAAG	78	ENSOART00000020945.1: 15–33
	R: GTCTGGTCCATGTTGTTCA		ENSOART00000020945.1: 74–92
*PRKAR2B*	F: CTCCAGTAATAAACCGATTTAC	110	ENSOART00000005398.1: 283–340
	R: GTCAGTTTTGGGATGTATAATC		ENSOART00000005398.1: 371–392
*ELOVL3*	F: TCGGTATCCTGGCTTATATC	117	ENSOART00000019158.1: 647–666
	R: GGAAGAACTTGACAAAGAGA		ENSOART00000019158.1: 744–763
*PDK4*	F: GGAACTGATGCTATCATCTA	81	ENSOART00000003809.1: 1066–1085
	R: GAAGGCTGATTTGTTAAAGA		ENSOART00000003809.1: 1127–1146
*PLIN2*	F: CTCAGGATAAGCTCTATCTG	73	ENSOART00000015469.1: 830–849
	R: TGGGATTCATCTGTATCATC		ENSOART00000015469.1: 883–902
*TCAP*	F: CTGCAGGAATACCAGCTG	189	ENSOART00000012585.1: 232–249
	R: CAGCTGCTTGGTGATCTC		ENSOART00000012585.1: 403–420
*SLC22A4*	F: ACCCAGACGTTATATCATAG	97	ENSOART00000016553.1: 1266–1285
	R: GATGGACAAGAAGTTGTAAC		ENSOART00000016553.1: 1343–1362
*LTF*	F: GCCATATAATTTCCATAATTTCATC	165	ENSOART00000009392.1: 4121–4145
	R: TTGGGTGTTTCAGAAAGTAA		ENSOART00000009392.1: 4266–4285
*ADGRG3*	F: GCTTGTTTCTCCTGAATCTG	176	ENSOART00000000666.1: 953–972
	R: GGTGTTAAAGACCTTGATGA		ENSOART00000000666.1: 1109–1128
*LEPR*	F: AAGGGTTCTATTTGTATTAGTGA	118	ENSOART00000011314.1: 2909–2931
	R: GGGTGGCATATTTAACAGAG		ENSOART00000011314.1: 3007–3026
*GAPDH*	F: CACTCACTCTTCTACCTT	91	NM_001190390.1: 900–917
	R: GCCAAATTCATTGTCGTA		NM_001190390.1: 973–990

**Table 2:** Primer pairs of differentially expressed lncRNAs used for qRT-PCR validation.

Name	Primer (5^′^→3^′^)	Product size (bp)	Primer position
*ENSOART00000027984*	F: CCAAGGGATTCTCAAGAG	113	ENSOART00000027984.1: 1031–1048
	R: GGTCTTCCAGTAGTCATG		ENSOART00000027984.1: 1126–1143
*ENSOART00000028008*	F: CTCTCCTTCCACAGAATC	144	ENSOART00000028008.1: 139–156
	R: GACCTGATGTATGCCAAG		ENSOART00000028008.1: 265–282
*ENSOART00000028118*	F: GTTCCTTTAGCCTCCTGA	76	ENSOART00000028118.1: 433–450
	R: CCACCTTGTCATCTTGAG		ENSOART00000028118.1: 491–508
*TCONS_00297891*	F: CAGGTATAAGCTAACTAGAAG	136	NC_019459.2: 55271478–55271498
	R: CACCCTTGCACTAATAAG		NC_019459.2: 55271596–55271613
*TCONS_00303998*	F: CAGTCCACTCAGAACAAC	194	NC_019459.2: 120843529–120843546
	R: CTTGGTGAACTATTCTTAGGA		NC_019459.2: 120843702–120843722
*TCONS_00616585*	F: CCACAAGAGGTATCTCAG	150	NC_019484.2: 93755689–93755706
	R: TCTCCATAGCTGCAATTAG		NC_019484.2: 93755820–93755838
*GAPDH*	F: CACTCACTCTTCTACCTT	91	NM_001190390.1: 900–917
	R: GCCAAATTCATTGTCGTA		NM_001190390.1: 973–990

Relative expressions were calculated using the 2^-ΔΔC_t_^ method with *GAPDH* as the internal control (Livak and Schmittgen, 2001). The data were compared by Student’s *t*-test using SPSS (version 23.0) (SPSS, Inc., Chicago, IL, United States), and the results were expressed as the mean ± standard deviation of triplicates values. *P*-value < 0.05 was considered statistically significant (Yang et al., 2017).

### Construction of the lncRNA-Gene Co-expression Network

To further explore the interactions between the DEGs and differentially expressed lncRNAs, the co-expression was analyzed based on their FPKM. For each lncRNA, Pearson correlation coefficient (COR) of its expression value with that of each mRNA was calculated. The interaction network of the differentially expressed lncRNA–mRNA co-expression pairs (an absolute value of COR ≥ 0.7 and FDR < 0.01) was then constructed using Cytoscape (v3.6.0) (Shannon et al., 2003).

## Results

### Sequencing Data Summary

Herein, a total of 60 Gb raw data were generated. In detail, 75,592,986, 88,617,414, and 83,525,778 raw reads were obtained for LFTS (LFTS-1, 2, and 3, respectively); 100,297,264, 80,848,034, and 83,364,558 raw reads were obtained for STHS (STHS-1, 2, and 3, respectively); and 78,883,006, 70,533,752, and 57,254,426 raw reads were obtained for TS (TS-1, 2, and 3, respectively) (**Table 3**). The raw reads were filtered to obtain clean reads, which were mapped to the *Ovis aries v3.1* version of the sheep genome sequence, with the mapping ratio ranging from 68.26 to 81.12%. Based on it, the transcripts were assembled using Cufflinks (v2.2.1) with default parameter. The results of the RNA-Seq reads mapped on the reference are shown in **Table 3**.

**Table 3:** Reads filter and mapping summary.

Sample ID	Raw reads	Clean reads	Clean ratio (%)	rRNA trimmed^∗^	rRNA ratio (%)	Mapped reads	Mapping ratio (%)
LFTS.1	75,592,986	62,250,244	82.35	62,084,744	0.27	50,361,708	81.12
LFTS.2	88,617,414	74,168,527	83.70	73,875,095	0.40	57,729,508	78.14
LFTS.3	83,525,778	65,120,752	77.96	64,996,175	0.19	48,378,447	74.43
STHS.1	100,297,264	85,223,789	84.97	84,220,736	1.18	59,272,741	70.38
STHS.2	80,848,034	66,088,476	81.74	65,911,628	0.27	52,818,507	80.14
STHS.3	83,364,558	71,214,729	85.43	71,075,656	0.20	56,545,968	79.56
TS.1	78,883,006	65,776,973	83.39	65,562,800	0.33	51,766,219	78.96
TS.2	70,533,752	53,370,250	75.67	52,620,265	1.41	35,919,719	68.26
TS.3	57,254,426	47,545,827	83.04	47,223,003	0.68	35,703,658	75.61

*^∗^rRNA trimmed reads are data which non-alignment to rRNA database of sheep. Clean ratio = (clean reads/raw reads)%; rRNA ratio = [(clean reads - rRNA trimmed)/clean reads]%; Mapping ratio = mapped reads/all reads*.

### Identification and Characterization of lncRNA in Tail Fat of Sheep

**FIGURE 1:**
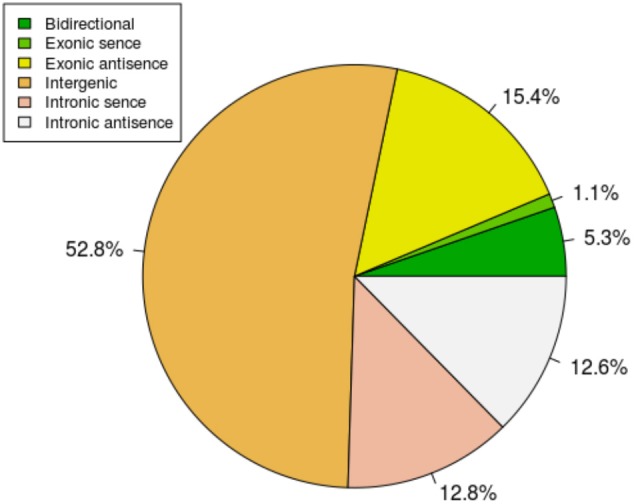
The classification of lncRNAs.

**FIGURE 2:**
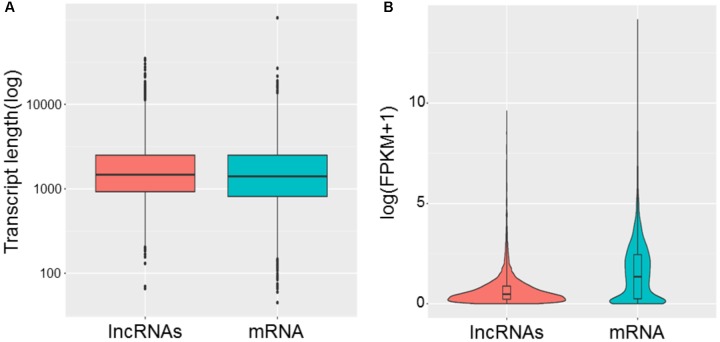
Comparison of length **(A)** and expression levels **(B)** between lncRNAs and mRNA.

To study the basic features of lncRNAs in tail fat of sheep, the lncRNAs were identified and compared with mRNA. The intersection of the Coding Potential Calculator, Coding-Non-Coding Index, and the Protein Families Database results finally yielded 9,082 lncRNA transcripts. The lncRNA transcripts were classified as 4,791 (52.8%) intergenic lncRNAs, 97 (1.1%) exonic sence lncRNAs, 1,398 (15.4%) exonic antisence lncRNAs, 1,167 (12.8%) intronic sence lncRNAs, 1,148 (12.6%) intronic antisence lncRNAs, and 481 (5.3%) bidirectional lncRNAs (**Figure 1**). Although the length of lncRNAs and mRNA transcripts is comparable, the expression levels between them are different. We found that lncRNAs exhibited lower expression levels compared to mRNA (**Figure 2**).

### Differential Expression Analysis and Target Gene Prediction

**FIGURE 3:**
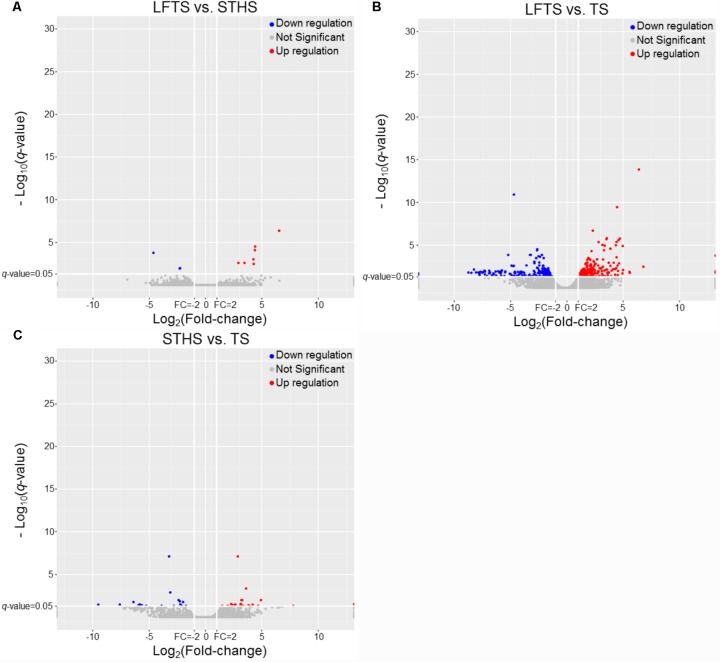
The volcano plot of gene expression levels in LFTS vs. STHS **(A)**, LFTS vs. TS **(B)**, and STHS vs. TS **(C)**. The vertical lines correspond to twofold up and down and the horizontal line represents a *q*-value of 0.05. The red point represents up-regulated DEGs, the blue point represents down-regulated DEGs, the gray point for no significant genes.

**FIGURE 4:**
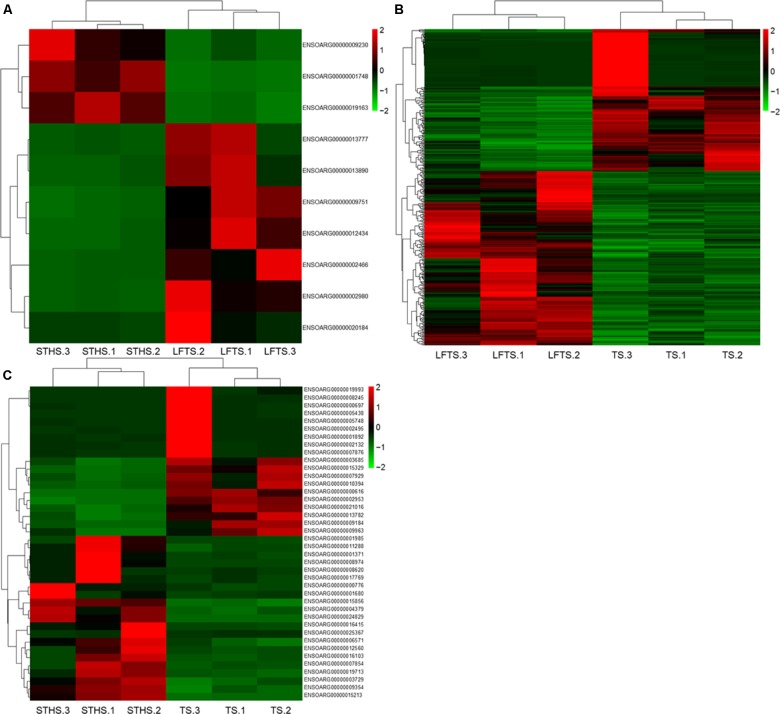
The hierarchical clustering of DEGs in LFTS vs. STHS **(A)**, LFTS vs. TS **(B)**, and STHS vs. TS **(C)**.

DEGs and differentially expressed lncRNAs were found through comparison between any two breeds. For the tail fat of LFTS vs. STHS, 10 genes were considered as DEGs, including 7 up-regulated and 3 down-regulated genes. For LFTS vs. TS, 390 genes were DEGs including 215 up-regulated and 175 down-regulated ones. For the comparison of STHS and TS, 40 DEGs were found of which 21 genes were up-regulated and 19 were down-regulated. The two common DEGs in LFTS vs. STHS and LFTS vs. TS were *FMO2* and *ENSOARG00000013777*. In total, 17 common DEGs were found in both LFTS vs. TS and STHS vs. TS groups, such as *C1RL, DHCR7*, and *IGF1*. There were no common DEGs in the two comparisons of LFTS vs. STHS and STHS vs. TS. We used volcano plots to explore the relationship between the fold change and the significance (**Figure 3**). To determine the primary patterns of gene expression, hierarchical clustering analysis of all DEGs was further employed based on the FPKM value (**Figure 4**).

**FIGURE 5:**
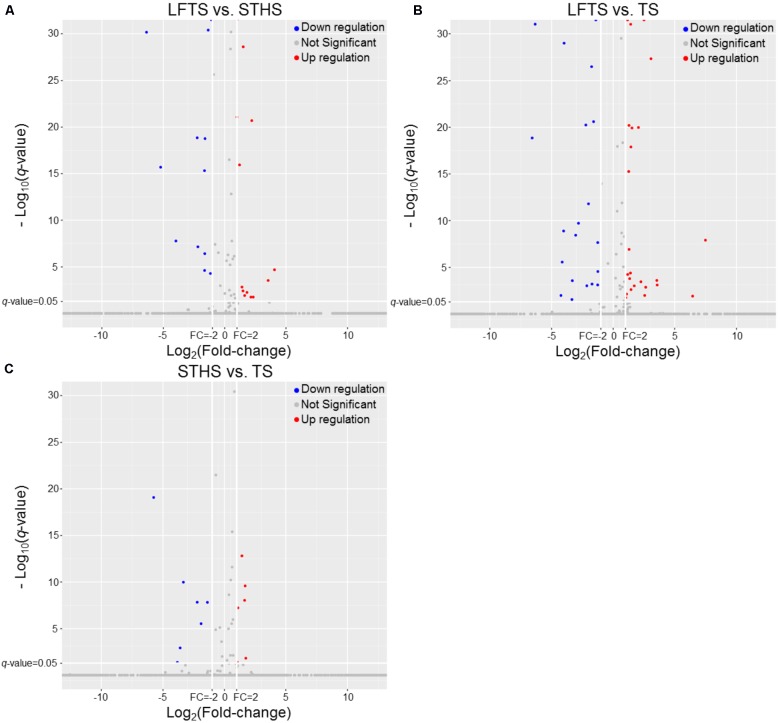
The volcano plot of lncRNAs expression levels in LFTS vs. STHS **(A)**, LFTS vs. TS **(B)**, and STHS vs. TS **(C)**. The vertical lines correspond to twofold up and down and the horizontal line represents a *q*-value of 0.05. The red point represents up-regulated differentially expressed lncRNAs, the blue point represents down-regulated differentially expressed lncRNAs, and the gray point for no significant lncRNAs.

By analysis, 68 differentially expressed lncRNAs were screened from the three comparisons. Among them, 37 differentially expressed lncRNAs (16 up-regulated and 21 down-regulated) were found between LFTS and STHS. Fifty-nine differentially expressed lncRNAs (31 up-regulated and 28 down-regulated) were found between LFTS and TS. There were 16 differentially expressed lncRNAs (eight up-regulated and eight down-regulated) between STHS and TS. The two common differentially expressed lncRNAs in the three comparisons were *TCONS_00297891* and *TCONS_00369087*. Except for these two lncRNAs, there were 27 common differentially expressed lncRNAs in the LFTS vs. STHS and LFTS vs. TS, 11 common differentially expressed lncRNAs in LFTS vs. TS and STHS vs. TS, and 2 common differentially expressed lncRNAs in LFTS vs. TS and STHS vs. TS. Volcano plots were used to explore the relationship between the fold change and the significance (**Figure 5**). As lncRNAs could exert effects through *cis*- or *trans*-acting target genes, the neighboring (100 kb upstream or downstream) and/or complementary protein-coding genes of the differentially expressed lncRNAs from pairwise comparisons were predicted.

### GO Analysis

**FIGURE 6:**
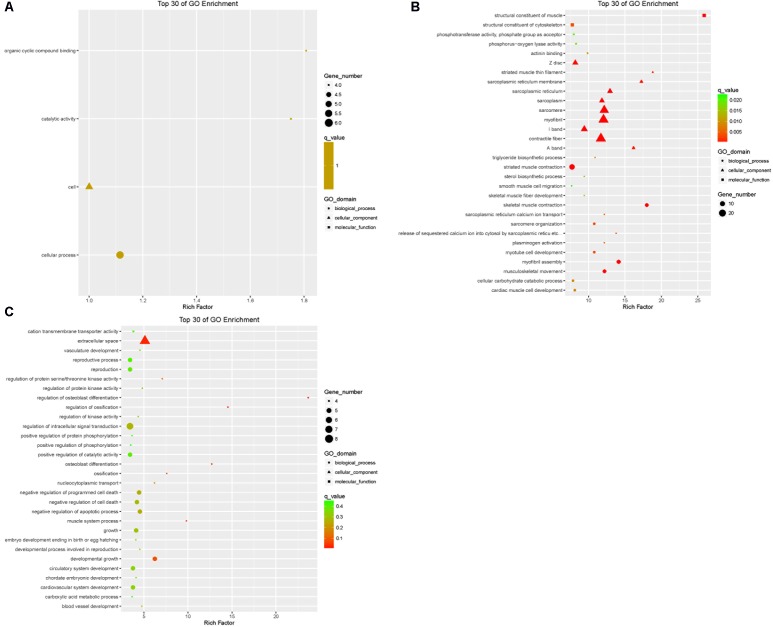
Top 30 of GO enrichment for DEGs from three groups of pairwise comparisons (**A**: LFTS vs. STHS, **B**: LFTS vs. TS, and **C**: STHS vs. TS). The *x*-axis presents rich factor of DEGs in a category. The *y*-axis shows the specific GO term.

The DEGs in the tail fat of LFTS vs. STHS, LFTS vs. TS, and STHS vs. TS were annotated (**Supplementary Table S2**). The top 30 GO terms (in descending order of the Richness factor) of the three groups are shown in **Figure 6**. The DEGs of LFTS vs. STHS were enriched in four GO terms, including organic cyclic compound binding, cell, catalytic activity, and cellular process. LFTS vs. TS DEGs were enriched in triglyceride biosynthetic process, sterol biosynthetic process, and cellular carbohydrate catabolic process. The DEGs of STHS vs. TS were majorly enriched in biological process including negative regulation of cell death and developmental growth.

**FIGURE 7:**
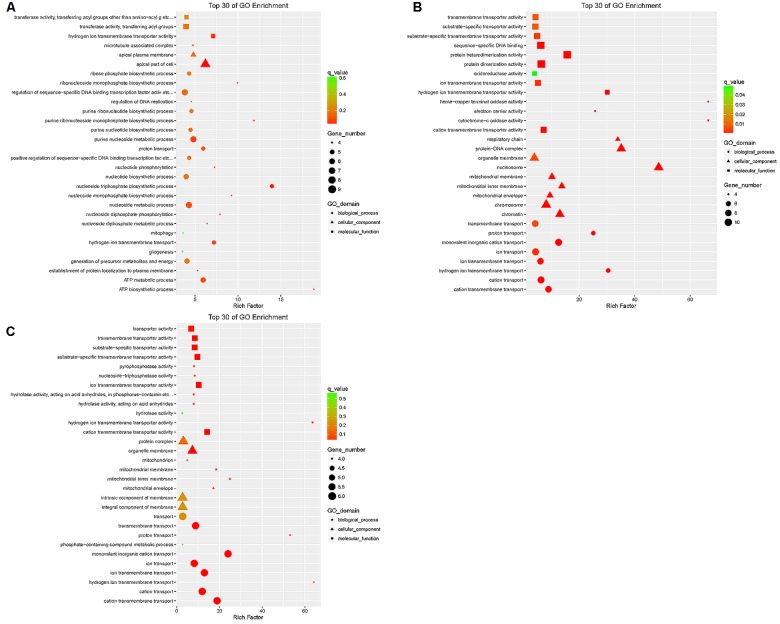
Top 30 of GO enrichment for target genes of differentially expressed lncRNAs from three groups of pairwise comparisons (**A**: LFTS vs. STHS, **B**: LFTS vs. TS, and **C**: STHS vs. TS). The *x*-axis presents rich factor of target genes in a category. The *y*-axis shows the specific GO term.

The target genes of differentially expressed lncRNAs in the tail fat of LFTS vs. STHS, LFTS vs. TS, and STHS vs. TS were annotated and the top 30 GO terms (in descending order of the Richness factor) of the three groups are shown in **Figure 7**. The target genes of LFTS vs. STHS were significantly enriched in four GO terms, including nucleoside triphosphate biosynthetic process, apical part of cell, ATP biosynthetic process, and purine ribonucleoside monophosphate biosynthetic process. LFTS vs. TS target genes were significantly enriched in 33 GO terms, such as protein–DNA complex, protein dimerization activity, and transporter activity. The target genes of STHS vs. TS were significantly enriched in 23 GO terms which mainly related to transporter activity and protein activity.

### Pathway Analysis

**FIGURE 8:**
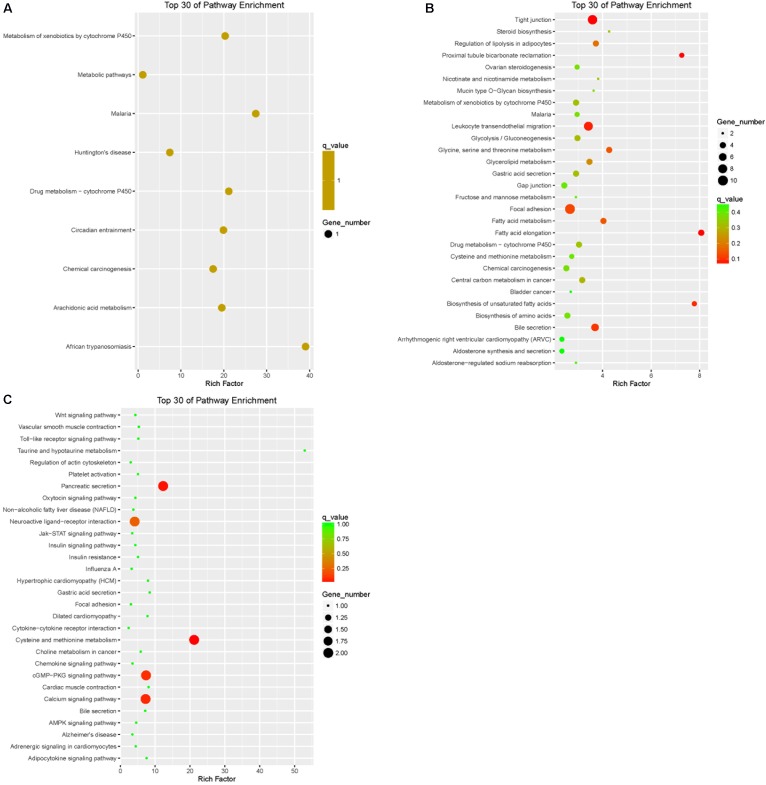
Top 30 of KEGG pathways enrichment for DEGs from three groups of pairwise comparisons (**A**: LFTS vs. STHS, **B**: LFTS vs. TS, and **C**: STHS vs. TS). The *x*-axis presents rich factor of DEGs in a category. The *y*-axis shows the specific pathway.

Pathway annotation of DEGs was performed using the KEGG database (**Supplementary Table S3**). Pathway enrichment analysis showed that the DEGs of LFTS vs. STHS related mainly to metabolic processes such as arachidonic acid metabolism and metabolism of xenobiotics by cytochrome P450; the DEGs of LFTS vs. TS were enriched in pathways including regulation of lipolysis in adipocytes, steroid biosynthesis, fatty acid metabolism, fatty acid elongation, and biosynthesis of unsaturated fatty acids; the pathways related to fat which the STHS vs. TS DEGs were enriched in included the adipocytokine signaling pathway, cGMP-PKG signaling pathway, and Jak-STAT signaling pathway (**Figure 8**).

**FIGURE 9:**
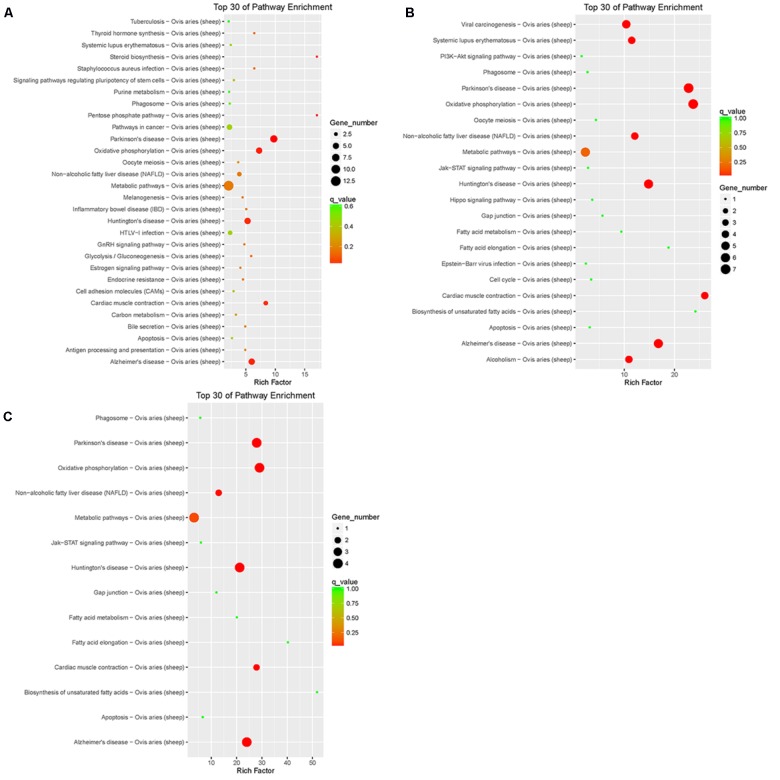
Top 30 of KEGG pathways enrichment for target genes of differentially expressed lncRNAs from three groups of pairwise comparisons (**A**: LFTS vs. STHS, **B**: LFTS vs. TS, and **C**: STHS vs. TS). The *x*-axis presents rich factor of target genes in a category. The *y*-axis shows the specific pathway.

Pathway annotation and enrichment of target genes of differentially expressed lncRNAs were performed using the KEGG database. The results showed that the target genes of differentially expressed lncRNAs of LFTS vs. STHS were majorly related to oxidative phosphorylation; the target genes of LFTS vs. TS were abundant in pathways including fatty acid elongation and fatty acid metabolism; and the pathways which the STHS vs. TS target genes were mainly enriched were in fatty acid elongation (**Figure 9**).

### Validation of RNA-Seq Data by qRT-PCR

**FIGURE 10:**
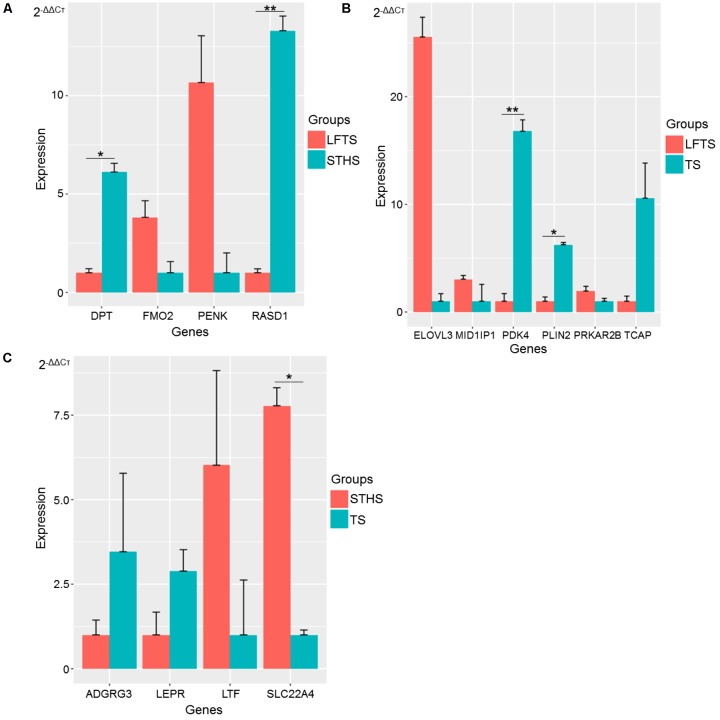
Validation of DEGs from three groups of pairwise comparisons by qRT-PCR (**A**: LFTS vs. STHS, **B**: LFTS vs. TS, and **C**: STHS vs. TS). The data presented in *y*-axis indicate genes expression as determined by qRT-PCR. ^∗^*p*-value < 0.05; ^∗∗^*p*-value < 0.01.

**FIGURE 11:**
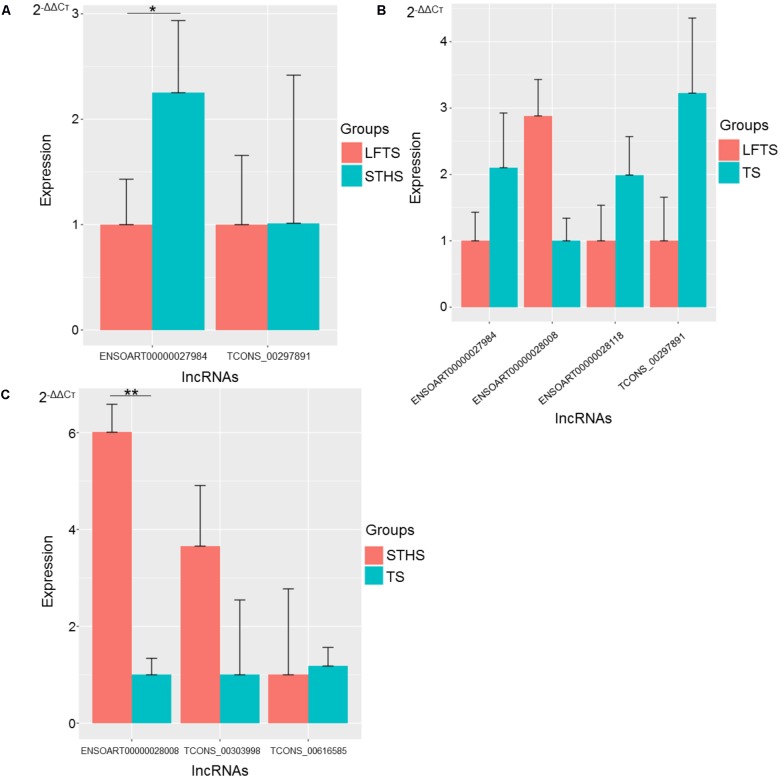
Validation of differentially expressed lncRNAs from three groups of pairwise comparisons by qRT-PCR (**A**: LFTS vs. STHS, **B**: LFTS vs. TS, and **C**: STHS vs. TS). The data presented in *y*-axis indicate lncRNAs expression as determined by qRT-PCR. ^∗^*p*-value < 0.05; ^∗∗^*p*-value < 0.01.

To validate the RNA-Seq data, DEGs and differentially expressed lncRNAs related to adipocyte accumulation were, respectively, selected in LFTS vs. STHS, LFTS vs. TS, and STHS vs. TS. In total, 14 and 6 DEGs and lncRNAs, respectively, underwent qRT-PCR analysis. The qRT-PCR results of the DEGs and differentially expressed lncRNAs were in agreement with the RNA-Seq data, indicating that the two results validated each other (**Figures 10**, **11**). Compared with STHS, the DEGs *FMO2* and *PENK* were up-regulated, whereas *DPT* and *RASD1* were down-regulated in the LFTS, where *DPT* showed significant differential expression (*p*-value < 0.05) and *RASD1* showed very significant differential expression (*p*-value < 0.01). Compared with TS, the DEGs *MID1IP1, PRKAR2B*, and *ELOVL3* were up-regulated, whereas *PDK4, PLIN2*, and *TCAP* were down-regulated in the LFTS, where *PLIN2* showed significant differential expression (*p*-value < 0.05) and *PDK4* showed very significant differential expression (*p*-value < 0.01). Compared with TS, the DEGs *SLC22A4* and *LTF* were up-regulated, whereas *ADGRG3* and *LEPR* were down-regulated in the STHS, where *SLC22A4* showed significant differential expression (*p*-value < 0.05).

For lncRNAs, compared with STHS, the differentially expressed lncRNAs *ENSOART00000027984* and *TCONS_00297891* were down-regulated in the LFTS, where *ENSOART00000027984* showed significant differential expression (*p*-value < 0.05). Compared with TS, the differentially expressed lncRNA *ENSOART00000028008* was up-regulated, whereas *ENSOART00000027984, ENSOART00000028118*, and *TCONS_00297891* were down-regulated in the LFTS. Compared with TS, the differentially expressed lncRNAs *ENSOART0 0000028008* and *TCONS_00303998* were up-regulated, whereas *TCONS_00303998* was down-regulated in the STHS, where *ENSOART00000028008* showed significant differential expression (*p*-value < 0.01).

The expression levels of these genes and lncRNAs as determined by qRT-PCR were consistent with the RNA-Seq data, which validated the accuracy of the RNA-Seq data.

### Network Construction Based on DEGs and Differentially Expressed lncRNAs in Tail Fat of Sheep

**FIGURE 12:**
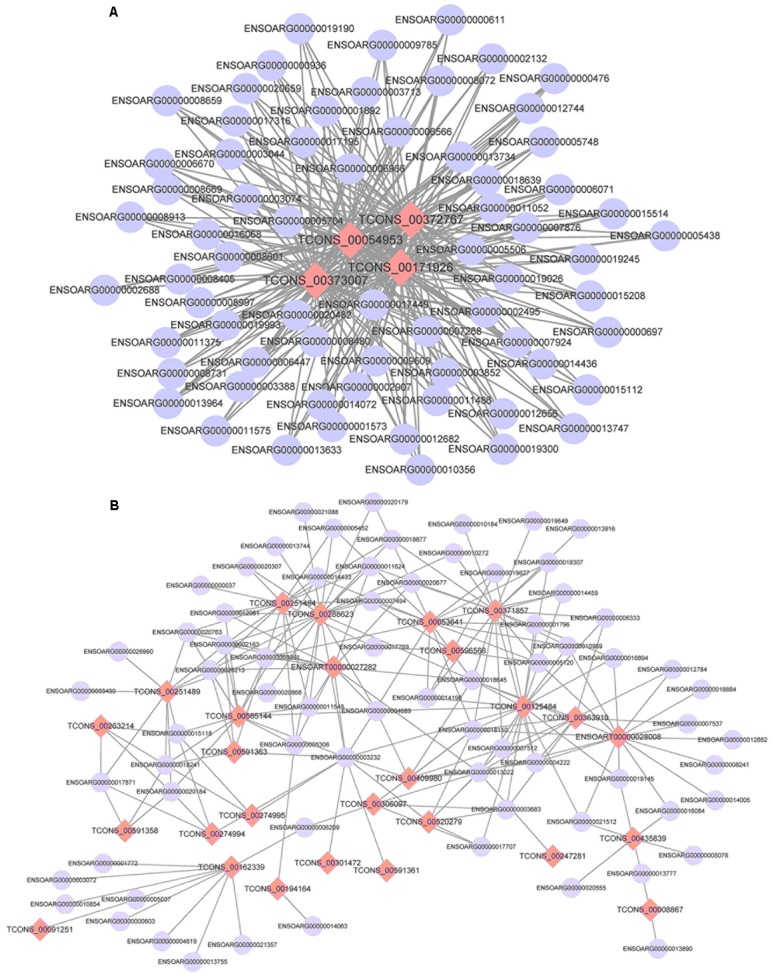
The co-expression networks of differentially expressed lncRNAs and DEGs (**A**: 4 lncRNAs and 67 mRNA and **B**: 27 lncRNAs and 71 mRNA).

Using the screened differential expression mRNA and lncRNA of tail fat of sheep for co-expression analysis, 493 pairs of significant co-expression pairs were obtained, and most were positively correlated (COR ≥ 0.7, 475 pairs) while a few were negatively correlated (COR ≤-0.7, 18 pairs). Using the screened mRNA–lncRNA pairs to construct a co-expression network, it was found that some lncRNAs interact with more than 50 mRNA, for example, 67 mRNA co-expressed with *TCONS_00372767, TCONS_00171926*, and *TCONS_00054953*, respectively, and 65 mRNA co-expressed with *TCONS_00373007*, indicating that these lncRNAs belong to the core lncRNAs and have important regulatory effects on tail fat deposition (**Figure 12**).

## Discussion

Transcriptome sequencing is the preferred biotechnique to analyze gene expression and reveal biological characteristics. Herein, we used tail fat from LFTS, STHS, and TS, which are unique Chinese sheep breeds, to explore the mechanism underlying the different tail phenotypes. Strand-specific RNA sequencing was performed to systematically identify mRNA and lncRNAs in different tail fat tissues. In this study, 407 DEGs were identified from the three comparison pairs and were significantly enriched in 120 GO terms and pathways. Furthermore, 68 differentially expressed lncRNAs were screened and the target genes of these lncRNAs were predicted. Further 493 significant co-expression pairs based on DEGs and differentially expressed lncRNAs were constructed to reveal their function.

We identified 9,082 lncRNAs from tail fat of LFTS, STHS, and TS, and most of them belong to intergenic lncRNAs. LncRNAs from tail fats are relatively abundant compared with these from other tissues, such as 6,924 and 5,602 lncRNAs from muscle and blood samples of Hu sheep, respectively (Zhang et al., 2017; Feng et al., 2018). The tail fat lncRNAs also share several typical characters with other mammalian lncRNAs. Compared with mRNA, the tail fat lncRNAs have relatively lower expression levels, while the length of lncRNAs was similar to that of mRNA. These similarities support that the lncRNAs identified in this study were reliable. To our knowledge, this study presents the first systematic genome-wide analysis of lncRNAs in tail fat of sheep, providing a valuable resource for functional lncRNAs associated with sheep tail fat deposition.

Of the 407 DEGs, a large proportion of key genes were involved in fat deposition, adipogenesis, and fatty acid biosynthesis, including *FMO2, PLIN2, PLIN3, LEPR, PENK, ELOVL3, ELOVL5, PDK4*, and *SLC22A4*.

Based on GO and pathway analyses of DEGs in LFTS and STHS, flavin-containing monooxygenases (FMOs) were enriched in four GO terms influencing fat metabolism. FMOs catalyze the NADPH-dependent oxidative metabolism of many structurally diverse foreign chemicals. Mice lacking FMOs 1, 2, and 4 exhibit a lean phenotype and despite similar food intake, weigh less and store less triglycerides in their white adipose tissue compared to wild-type mice (Veeravalli et al., 2014). *FMO2* and *FMO3* are members of the *FMO* gene family and *FMO3* was identified by a recent comparative genomic study between fat- and thin-tail sheep using RNA-Seq data with respect to adipose tissues from Wang et al. (2014).

Through GO enrichment of LFTS vs. TS, DEGs enriched in fatty acid elongation, biosynthesis of unsaturated fatty acids, and fatty acid biosynthesis pathways were found to be up-regulated. Previous studies have shown that breed effect was significant on fatty acid composition of fat tail (Unsal and Aktas, 2003; Moharrery, 2007; Alipanah and Kashan, 2011). Four DEGs were enriched in the triglyceride biosynthetic process including three up-regulated genes (*PCK1, GPAM*, and *LDLR*). This could indicate that the fat accumulation of LFTS was more than that in TS, leading to rapid fat metabolism. Moreover, *ELOVL3, ELOVL5, PLIN2, PLIN3, NR4A1*, and *KLF4* genes were differentially expressed between LFTS vs. TS. *ELOVL, PLIN*, and *KLF* gene families were identified to be possibly associated with tail fat deposition (Miao et al., 2015b). *NR4A1* and *KLF7* were reported to be associated with adipocyte differentiation (Duszka et al., 2012; Zhang Z. et al., 2013). This suggested that these DEGs are possibly related to fat deposition in the tails of sheep.

In the comparative analysis of STHS and TS, the GO enrichment term “negative regulation of cell death” was focused on. Among the DEGs, *IGF1, SERP2*, and *CITED1* were up-regulated, whereas *ALB* and *ACTC1* were down-regulated in STHS. The other GO term was related to growth and included up-regulated genes (*NPK, SERP2, DHCR7*, and *IGF1*) in STHS. *IGF1* stimulates both the proliferation and differentiation of pre-adipocytes in cell culture (Duffield et al., 2008). Furthermore, *CITED1* gene promotes cell proliferation and migration, and it is also a marker gene when browning of white adipocytes was induced (Choi et al., 2018; Xia et al., 2018). In addition, *SLC22A4* was differentially expressed between STHS and TS, and *SLC27A6* was identified as a candidate gene in tail fat development (Kang et al., 2017). *SLC22A4* and *SLC27A6* have similar functions. This suggests that *SLC22A4* genes are possibly related to the fat-tail dimensions in sheep.

In this study, 68 differently expression lncRNAs were identified and the target genes of these lncRNAs were predicted. The results showed that the target genes were principally enriched in the GO term associated with mitochondria and transmembrane transport, such as mitochondrial inner membrane and transporter activity. The target genes also were mostly enriched in oxidative phosphorylation and non-alcoholic fatty liver disease (NAFLD). The most commonly enriched target genes were *ATP6, ATP8, COIII, COXl, COX2, FHLl, SLC24A2, ALDOA*, and *ND1*. ATP plays an important role in adipocyte. ATP could release energy to produce ADP and inorganic phosphate (Pi). AMP-activated protein kinase (AMPK) controls a constant high ratio of ATP to ADP (Hardie, 2011). The fatty acids produced by lipolysis are not usually oxidized within the adipocyte, but are released for use elsewhere. If the fatty acids generated by lipolysis are not rapidly removed from adipocytes either through export or by oxidative metabolism, they are recycled into triglycerides, an energy intensive process in which two molecules of ATP are consumed per fatty acid (Hardie, 2012). Thus, AMPK could inhibit lipolysis and maintain the rate of ATP to ADP. However, the different tail fat were used according to the condition of different sheep and the amount of fat deposition. Another special target is *ELOVL6*, which is found between the LFTS vs. TS comparison and is associated with fatty acids. Interestingly, the DEGs of LFTS vs. TS included *ELOVL3* and *ELOVL5*. It could indicate that the *ELOVL* genes are differently expressed and regulated between tail fats of LFTS and TS that the characters are relatively different.

A total of 493 pairs of co-expression pairs were obtained by network construction based on DEGs and differentially expressed lncRNAs in tail fat of sheep. Among these co-expressed pairs, most of them were significantly and positively correlated, and only a small pairs are negatively correlated. These results indicate that these mRNA and lncRNAs may play a role mainly through positive regulation. That is high expression or low expression of both. It was also found that some lncRNAs can be co-expressed with many mRNA, suggesting that may be the lncRNAs were regulated by many mRNA.

The regulation of lipogenesis is a very complex biological process, and the tail fat of sheep is no exception. Previous studies have reported that tail fat development in sheep is associated with mRNA and miRNA (Wang et al., 2014; Miao et al., 2015a,b; Kang et al., 2017; Li et al., 2018b; Pan et al., 2018). These studies also show that tail fat deposition in sheep is not only regulated by a gene or miRNA, more likely by many coding and non-coding RNA. Some researchers integrated the miRNA and mRNA from Kazakhstan sheep and TS and found that the miRNA can participate in the regulation of sheep fat deposition by target mRNA (Zhou et al., 2017). As a type of non-coding RNA, lncRNA can also participate in the regulation of fat as part of a competing endogenous RNA network. From the perspective of lncRNAs, this study speculated that it regulates the tail fat deposition of sheep based on the lncRNA–mRNA regulated network.

In addition, there are some shortcomings in this study. For example, the DEGs and differentially expressed lncRNAs were to some extent caused by breed effect. Moreover, three animals per group are statistically not powerful enough. Regardless of the technology used to measure expression levels and the size of samples, the true gene expression levels will vary among individuals because expression is inherently a stochastic process (Hansen et al., 2011). In that case, the analysis results may not be powerful enough. However, the biological variability decreases with the increase of the number of samples. Hence, we hope to go on the further study with a larger sample size in the near future.

## Conclusion

A total of 407 DEGs and 68 differentially expressed lncRNAs were identified between LFTS, STHS, and TS tail fat tissues (*q*-value < 0.05), among which were potentially associated with tail adipose tissue enlargement. These findings contribute to a better understanding of adipose deposits in regulating the regional fat distribution and the diverse tail types in fat-tailed sheep breeds.

## Author Contributions

XL and YC conceived the project and designed the experiments. HC provided suggestions for the project. LM analyzed the data as well as he drafted the manuscript under the supervision of XL. YC and LH collected sheep tail fat tissue samples. YJ and SE performed the RNA extraction. LM and MZ performed qRT-PCR.

## Conflict of Interest Statement

The authors declare that the research was conducted in the absence of any commercial or financial relationships that could be construed as a potential conflict of interest.

## Footnotes

1 https://github.com/lh3/seqtk

2 http://www.geneontology.org/

3 http://www.genome.jp/kegg/

## Supplementary Material

TABLE S1 Quality assessment reports of all RNA samples.

TABLE S2 The GO annotation of DEGs from three groups of pairwise comparisons.

TABLE S3 The KEGG annotation of DEGs from three groups of pairwise comparisons.
